# Supplementary material for: From sequence to enzyme mechanism using multi-label machine learning
Source: BMC Bioinformatics. 2014 May 19;15:150. doi: 10.1186/1471-2105-15-150 (PMC4229970; doi:10.1186/1471-2105-15-150)
Supplement: Additional file 2 — Java code of ml2db. Additional file ml2db_code.tar.gz contains the Java source code to run the multi-label machine learning experiments and save the results to database. The code’s Javadoc is included. [file 1471-2105-15-150-S2.zip › additional file 2/ml2db/ml2db/doc/index-files/index-24.html]

X-Index


JavaScript is disabled on your browser.


- Overview
- Package
- Class
- Use
- Tree
- Deprecated
- Index
- Help

- Prev Letter
- Next Letter

- Frames
- No Frames

- All Classes

A B C D E F G H I J K L M N O P Q R S T U V W X 


## X

XML\_DATA\_PROP - Static variable in class main.ml2db.weka.ArffProps


XML\_FILE\_PATH\_PROP - Static variable in class main.ml2db.weka.ArffProps
:   needed for compatibility with Mulan arff files (which have 1 arff file +
    1 xml labels file)

XML\_QUERY - Static variable in class test.ml2db.dataharness.ArffPropsQueriesOneTest


XML\_QUERY - Static variable in class test.ml2db.dataharness.ArffPropsQueriesTwoTest


XML\_QUERY\_PROP - Static variable in class main.ml2db.mulan.mulanarff.MulanArffProperties
:   The query to get the class labels for the Mulan xml file (if needed)

A B C D E F G H I J K L M N O P Q R S T U V W X

- Overview
- Package
- Class
- Use
- Tree
- Deprecated
- Index
- Help

- Prev Letter
- Next Letter

- Frames
- No Frames

- All Classes
